# On the Properties of the Reaction Counts Chemical Master Equation

**DOI:** 10.3390/e21060607

**Published:** 2019-06-19

**Authors:** Vikram Sunkara

**Affiliations:** 1 Computational Medicine, Zuse Institute Berlin, 14195 Berlin, Germany; sunkara@mi.fu-berlin.de; 2 Department of Mathematics and Computer Science, Freie Universität Berlin, 14195 Berlin, Germany

**Keywords:** chemical master equation, jump continuous-time Markov chains, reaction counts

## Abstract

The reaction counts chemical master equation (CME) is a high-dimensional variant of the classical population counts CME. In the reaction counts CME setting, we count the reactions which have fired over time rather than monitoring the population state over time. Since a reaction either fires or not, the reaction counts CME transitions are only forward stepping. Typically there are more reactions in a system than species, this results in the reaction counts CME being higher in dimension, but simpler in dynamics. In this work, we revisit the reaction counts CME framework and its key theoretical results. Then we will extend the theory by exploiting the reactions counts’ forward stepping feature, by decomposing the state space into independent continuous-time Markov chains (CTMC). We extend the reaction counts CME theory to derive analytical forms and estimates for the CTMC decomposition of the CME. This new theory gives new insights into solving hitting times-, rare events-, and a priori domain construction problems.

## 1. Introduction

Continuous-time Markov chains (CTMC) accurately capture the dynamics of a broad range of biochemical reaction systems. The CTMCs come in two flavours: The discrete state space, the chain transitions from one state to another, or the continuous state space, the state transitions are smooth yet non-differentiable. Each type has an intuitive interpretation. Discrete state spaces describe the population or counts of chemicals in a system, while continuous states spaces describe concentrations of chemicals in a system [1,2]. It is tempting to generalise concentrations to be simply scaled populations, which would mean that all systems could be mapped into the continuous setting and solved there. However, this is not the case mathematically. It was shown that for large populations, the discrete CTMC and continuous CTMC were equivalent [3,4]. If the large population assumption is not satisfied, mathematically, the discrete state space system has to be studied in its own right.

The probability distribution of a CTMC over the discrete state space in the biochemical context is found by solving the chemical master equation (CME). We first present the CME and then describe its components. The formula of the CME is written as follows,

(1) $$\frac{\partial p(Z(t)=z)}{\partial t}=\sum_{n=1}^{N_{r}}a_{n}\left(z-\nu_{n}\right)\;p\left(Z\left(t\right)=z-\nu_{n}\right)-\sum_{n=1}^{N_{r}}a_{n}\left(z\right)\;p\left(Z\left(t\right)=z\right).$$

In the equation above, Z(t) is a CTMC over the state space $\Omega \subset N_{0}^{N_{s}}.$ Each state is a vector of $N_{s}\in N$ non-negative integers describing the population of the species at time t≥0. Starting from the left-hand side, the equation states that the change in probability of being in a state z∈Ω at time *t* is given by the sum of two terms. The first term describes the probability flowing into the state z and the second term describes the probability flowing out of the state z. There are $N_{r}\in N$ reactions perturbing the population; the terms $\nu_{n}$ are referred to as stoichiometric vectors, they capture the net change in population if the *n*th reaction fires. Hence, subtracting the stoichiometric vectors $\nu_{n}$ from z gives us the set of all previous states which might transition into z and “contribute” probability. The last piece of information in the equation are the rates at which reactions fire, these are encapsulated in the propensity functions $\{a_{n}:\Omega \to R_{+},\;\mathrm{for}\;n=1,\ldots ,N_{r}\}.$ In summary, the CME describes the change in probability of observing a CTMC in a certain state, which is equal to the sum of probability transitioning into the state, minus the sum of probability leaving the state.

Even though the dynamics of the derivative are fairly simple, solving the CME is a hard problem [5,6,7,8,9,10,11,12]. A special case of the CME which is truly unyielding to approximation is when a system is close to its population boundary (for example, close to zero). In this scenario, none of the state transitions around a state near the boundary can be summarised by the average transitions out of the state. This impedes most approximation methods, and even if a method was successful, its computational effort would be equivalent to that of solving the Finite State Projection for the same error. An approach which was proposed in various different contexts to simplify dynamics, was to study the number of reactions fired rather than the population counts [6,13,14,15,16,17]. Because reactions cannot “un-fire”, the state transitions are only moving forward. Furthermore, given that the system’s starting population is known, the species count in any state can be reconstructed from the reaction counts.

In this work, we will reintroduce the reaction counts variant of the CME. We will then explore the theoretical and structural results which emerge from its forward stepping process. Then, we will show how the reaction counts CME can be used to construct solutions to the classical CME. Lastly, we decompose the state space of the CME into independent CTMCs and give analytical forms and estimates to calculate their probabilities. The purpose of this work is to gain intuition and explore structural properties of the CME, hence, the motivation is more theory rather than the application of the CME. In light of that, we omit a discussion of numerical methods in this paper and envisage this aspect in future research.

## 2. Reaction Counts CME

### 2.1. Formulation

We denote the state space of the reaction counts CME by $\Lambda \subset N_{0}^{N_{r}}.$ Each state is a vector of non-negative integers of length $N_{r}.$ Each element of the vector represents the number of times its corresponding reaction has fired. In the CME setting, the change in state after a reaction fires is given by the stoichiometric vector, which we denoted earlier by $v_{n},$ for $n=\{1,\ldots ,N_{r}\}.$ This vector quantifies the net change in populations after the reaction has fired. Analogously, in the reaction counts CME setting, a change in state indicates that a reaction has fired, and its corresponding reaction count is incremented by one. Therefore, the stoichiometric vector for the *n*th reaction is simply the identity vector $1_{n};$ the vector is zero in all but the *n*th position, where it is one.

We bridge the species counts CME and the reaction counts CME with the mapping Γ:Ω×Λ→Ω, that is, for $(x_{0},r)\in \Omega \times \Lambda ,$

(2) $$\begin{array}{ccc} \Gamma (x_{0},r) & := & x_{0}+\left[v_{1},v_{2},\ldots ,v_{N_{r}}\right]\;r,\\ & = & x_{0}+\sum_{n=1}^{N_{r}}v_{n}\;r_{n}. \end{array}$$

The mapping above links the reaction counts CME to the species counts CME by stating that, given the starting state $x_{0}$ and the reactions which have fired are known, then the current state in the species counts is the starting species state plus the sum of stochiometries of all the reactions which have fired. It is important to note that the map Γ is injective into the species state space Ω. In most cases, Λ is of higher dimension than Ω, hence, Γ is seldom bijective. For our purpose, we only need the pull back map of Γ. We denote the pull back map as $\Gamma^{-1}:\Omega \times \Omega \to P\left(\Lambda \right),$ where for $(x_{0},x)\in \Omega \times \Omega ,$

(3) $$\Gamma^{-1}\left(x_{0},x\right):=\left\{r\in \Lambda :\Gamma \left(x_{0},r\right)=x\right\}.$$

With the mapping between Λ and Ω established, we can now inherit the propensity function from the species counts over to the reaction counts. For $n=1,\ldots ,N_{r},$ the reaction counts propensity of the *n*th reaction is given by,

(4) $$\alpha_{n}\left(x_{0},r\right):=\left\{\begin{array}{cc} a_{n}\left(\Gamma \left(x_{0},r\right)\right) & \mathrm{if}\;\Gamma (x_{0},r)\in \Omega ,\\ 0 & \mathrm{otherwise}. \end{array}\right.$$

With the state space, stoichiometry, and the propensities established in the reaction counts setting, we are ready to formulate the reaction counts based Kurtz process. For $x_{0}\in \Omega ,$ let ${\left(R_{x_{0}}\left(t\right)\right)}_{t\geq 0}$ be the reaction counts Kurtz process,

(5) $$R_{x_{0}}\left(t\right):=\sum_{n=1}^{N_{r}}1_{n}\;P\left(\int_{0}^{t}\alpha_{n}\left(x_{0},R_{x_{0}}\left(s\right)\right)ds\right).$$

The corresponding reaction counts CME for the process above is given by,

(6) $$\frac{dp(R_{x_{0}}\left(t\right)=r)}{dt}=\sum_{n=1}^{N_{r}}\alpha_{n}\left(x_{0},r-1_{n}\right)\;p\left(R_{x_{0}}\left(t\right)=r-1_{n}\right)-\sum_{n=1}^{N_{r}}\alpha_{n}\left(x_{0},r\right)\;p\left(R_{x_{0}}\left(t\right)=r\right),$$

for all r∈Λ. The initial condition for the reaction counts CME is a points mass on the origin,

(7) $$p\left(R_{x_{0}}\left(0\right)=r\right)=\left\{\begin{array}{cc} 1 & \mathrm{if}\;r=0,\\ 0 & \mathrm{otherwise}. \end{array}\right.$$

Upon first glance, the CME of the reaction counts is simpler in its complexity compared to its species counter part. In the reaction case, the processes only move forward, which gives rise to simple forward propagating dynamics. Furthermore, given solutions to the reaction counts CME exist, with the simple application of the push forward measure, we would obtain:

(8) $$p\left(X\left(t\right)=x\right)=\sum_{r\in \Gamma^{-1}\left(X\left(0\right),x\right)}p\left(R_{X(0)}\left(t\right)=r\right).$$

The relationship given above is the critical motivation for studying the reaction counts CME. In principle, if structures and results could be attained in the reaction counts setting, then by using Γ, these results can be mapped into species setting. The simplest example of this is the proof for the existence of solutions of the CME for finite time. We now show that the reaction counts CME has analytical solutions, then using the push forward measure in Equation (8), we prove that the solutions of the CME exist for a broad range of propensity functions.

### 2.2. Analytical Solutions of the Reaction Counts CME

Firstly, we will establish the notations and assumptions needed to present the results. For brevity, we denote the sum of all propensities $\alpha_{n}$ as:

(9) $$\alpha \left(\cdot ,r\right):=\sum_{n=1}^{N_{r}}\alpha_{n}\left(\cdot ,r\right).$$

The reaction counts CME in Equation (6) is a difference differential equation, hence, by arranging the probability states in a vector $u\left(t\right):={\left(p\left(R_{x_{0}}\left(t\right)=r\right)\right)}_{r\in \Lambda },$ we reduce solving the reaction counts CME to solving the ODE,

$$\frac{du(t)}{dt}=A^{*}\;u\left(t\right).$$

For r,l∈Λ, the matrix $A^{*}$ has the properties: ${\left[A^{*}\right]}_{r,r}\leq 0,$${\left[A^{*}\right]}_{r,l}\geq 0,$ with $\sum_{l\in \Lambda }{\left[A^{*}\right]}_{l,r}=0.$ We will prove that $A^{*}$ is a generator of a one-parameter semigroup. Before this, let us consider a simple two reaction system to gain some intuition into the different components of the reaction counts setting.

**Example** **1.** *Let us consider the birth-death process in the context of the reaction counts CME. The birth-death process in the species count is given by,*

$$Z\left(t\right)=x_{0}+P\left(\int_{0}^{t}c_{b}\;ds\right)-P\left(\int_{0}^{t}c_{d}\;Z\left(s\right)\;ds\right),$$

*where $x_{0}\in N_{0}$ is the starting population, and the birth and death rates are denoted by $c_{b}$ and $c_{d},$ respectively. We can translate this process into the reaction counts setting using the mapping,*

$$\Gamma_{x_{0}}\left(r_{1},r_{2}\right)=x_{0}+r_{1}-r_{2}.$$

*We restrict the reaction counts state space $\Lambda \subset N_{0}\times N_{0}$ to only contain states which yield non-negative values by applying $\Gamma_{x_{0}}.$ Substituting the mapping $\Gamma_{x_{0}}$ and the process Z(t) into Equations *(2)* to *(5)* leads to the reaction counts birth-death process formulation:*

(10) $$R_{x_{0}}\left(t\right)={\left(\begin{array}{c} 1\\ 0 \end{array}\right)}^{T}\;P\left(\int_{0}^{t}c_{b}\;ds\right)+{\left(\begin{array}{c} 0\\ 1 \end{array}\right)}^{T}\;P\left(\int_{0}^{t}c_{d}\;\Gamma_{x_{0}}\left({\left(R_{x_{0}}\left(s\right)\right)}_{1},{\left(R_{x_{0}}\left(s\right)\right)}_{2}\right)\;ds\right).$$

**Figure 1:**
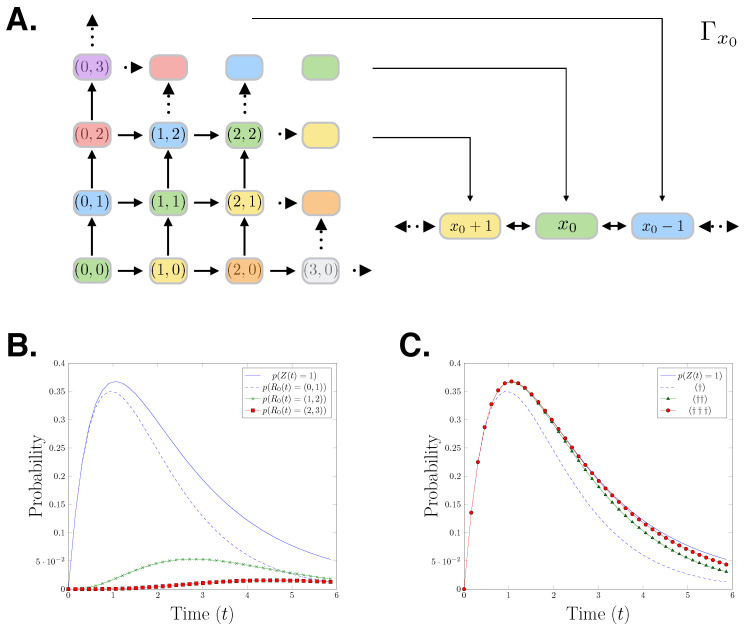
(**A**) Cartoon showing the mapping of the reactions counts birth-death process to the species count birth-death process. (**B**) An evaluation of the birth-death process for parameters $(x_{0}=0,c_{b}=1.0,c_{d}=0.1).$ The plot shows: The distribution of the species having population one over time p(Z(t)=1); and the distributions of reaction (1,0),(1,2), and (2,3) firing at time t, in the reaction counts setting. (**C**) $\left(†\right)=p(R_{0}\left(t\right)=\left(0,1\right))$, $\left(††\right)=p\left(R_{0}\left(t\right)=\left(0,1\right)\right)+p\left(R_{0}\left(t\right)=\left(1,2\right)\right)$, $\left(†††\right)=p\left(R_{0}\left(t\right)=\left(0,1\right)\right)+p\left(R_{0}\left(t\right)=\left(1,2\right)\right)+p\left(R_{0}\left(t\right)=\left(2,3\right)\right).$ The distribution of the species count chemical master equation (CME) in state 1 at *t* is approached from below by the sum of the probabilities of the reactions firing which result in being in state 1.

*The relationship between the reaction count state space and the species count state space is visualised in Figure 1A. The colours in the figure show how the species count state space partitions the reaction count state space. To demonstrate the forward moving structure of the reaction counts CME, we derive the generator for the first nine states of its state space:*

$$\frac{du(t)}{dt}=A^{*}\;u\left(t\right),$$

*where,*

$$u\left(t\right)={\left(\frac{dp(R_{x_{0}}\left(t\right)=\left(0,0\right))}{dt},\frac{dp(R_{x_{0}}\left(t\right)=\left(1,0\right))}{dt},\frac{dp(R_{x_{0}}\left(t\right)=\left(0,1\right))}{dt},\ldots \right)}^{T},$$

$$A^{*}=[\begin{array}{ccccccc} \frac{\left(0,0\right)}{-c_{b}-c_{d}x_{0}} & \frac{\left(1,0\right)}{0} & \frac{\left(0,1\right)}{0} & \frac{\left(2,0\right)}{0} & \frac{\left(1,1\right)}{0} & \frac{\left(0,2\right)}{0}\\ c_{b} & -c_{b}-c_{d}\left(x_{0}+1\right) & 0 & 0 & 0 & 0 & \ldots \\ c_{d}x_{0} & 0 & -c_{b}-c_{d}\left(x_{0}-1\right) & 0 & 0 & 0 & \ldots \\ 0 & c_{b} & 0 & -c_{b}-c_{d}\left(x_{0}+2\right) & 0 & 0 & \ldots \\ 0 & c_{d}\left(x_{0}+1\right) & c_{b} & 0 & -c_{b}-c_{d}x_{0} & 0 & \ldots \\ 0 & 0 & c_{d}\left(x_{0}-1\right) & 0 & 0 & -c_{b}-c_{d}\left(x_{0}-2\right) & \ldots \\ \vdots & \vdots & \vdots & \vdots & \vdots & \vdots & \ddots \end{array}].$$

The initial condition is the identity vector with the total probability mass on state (0,0). We can observe that due to the forward moving nature of the reaction counts setting, $A^{*}$ is a lower diagonal matrix. In Figure 1B,C, we present a birth-death process with initial state $x_{0}=0,$ birth rate $c_{b}=1.0,$ and death rate $c_{d}=0.1.$ Specifically, we show the probability distributions of the first three states of that process which correspond to state 1 in the species count setting. In Figure 1B, it is shown that the probability distribution of being in state 1 in the species count CME setting bounds the corresponding reaction counts CME distributions. In Figure 1C, we progressively add the first three reaction counts states corresponding to the state 1, *showing that the reaction counts CME approximates the species counts CME from below*.

**Theorem** **1.** (Sunkara 09 ([15])) *Given a system with $N_{s}$ species and $N_{r}$ reactions at a starting state $x_{0}\in \Omega .$ If $A_{r}$ is the generator for the reaction counts CME *(6)*, then:*

*1*. There exists a permutation matrix P such that $P^{T}\;A^{*}\;P$ is lower triangular;
*2*. The spectrum of $A^{*}$ is the set:
  (11) $$spec\left(A^{*}\right)=\left\{-\sum_{n=1}^{N_{r}}\alpha_{n}\left(x_{0},r\right):r\in \Lambda \right\}.$$

The property of the reaction counts setting, that the process only moves forward, aids in showing that the generator could be rewritten as a lower triangular matrix. We know that the spectrum of a lower triangular matrix are the diagonal elements of the matrix. In our case, the diagonal elements are simply the negative sum of the outgoing propensities. This then reveals that the spectrum of the generator $A^{*}$ is in the negative real numbers. Then by the spectral mapping theorem, solutions to the reaction counts CME exist. We can further exploit this “forward stepping” structure to write the analytical solutions for the probability at each state in Λ.

**Proposition** **1.** (Sunkara 09 ([15])) *Given a system with $N_{s}$ species and $N_{r}$ reactions at a starting state $x_{0}\in \Omega .$ Let C∈(0,1]. If ${\left(R_{x_{0}}\left(t\right)\right)}_{t\geq 0}$ is the reaction counts Kurtz process *(5)* with Λ≠∅, then the solution to *(6)* at t>0 is given by:*

(12) $$p\left(R_{x_{0}}\left(t\right)=0\right)=e^{-\alpha (x_{0},r)\;t}\;C,$$

*for r=0, and,*

(13) $$p\left(R_{x_{0}}\left(t\right)=r\right)=\sum_{n=1}^{N_{r}}\left(\alpha_{n}\left(x_{0},r-1_{n}\right)\;\int_{0}^{t}e^{-\alpha \left(x_{0},r\right)\;\left(t-s\right)}\;p\left(R_{x_{0}}\left(s\right)=r-1_{n}\right)ds\right),$$

*for r∈Λ\0.*

Combining Proposition 1 with the push forward measure in Equation (8), we see that at finite time *t* the solutions to the CME (1) exist. Unfortunately, since the reaction counts CME cannot reach a stationary state (in cases where the state space Λ is not finite), the proof for the existence of stationary solutions for a generalised CME is still an open problem. Now that the solutions of the reaction counts CME have been established, we can probe further into its properties. Firstly, we explore how the state space of the reaction count CME has a naturally hierarchical partitioning, and how this partitioning can be used to build a sequence of approximate sub-processes.

## 3. Partitioning the State Space

**Definition** **1.** *For m∈N, we define,*

$$\nabla_{m}:=\left\{r\in \Lambda :{∥r∥}_{1}=m\right\},$$

*to be the set of all states which are reachable by m steps from the origin 0. The state space *Λ* naturally partitions into non-intersecting subsets,*

$$\Lambda =\nabla_{0}\cup \nabla_{1}\cup \nabla_{2}\cup \ldots .$$

If we consider truncating the state space progressively, we find that this is equivalent to the Finite State Projection with an *N*-step domain expander [9,18].

**Lemma** **1.** *For $x_{0}\in \Omega ,$ let ${\left(R_{x_{0}}\left(t\right)\right)}_{t\geq 0}$ be the reaction counts Kurtz process *(5)*. Then for any m∈N,*

(14) $$\sum_{r_{1}\in \nabla_{m}}\sum_{n=1}^{N_{r}}\alpha_{n}\left(x_{0},r_{1}\right)\;\int_{0}^{t}p\left(R_{x_{0}}\left(s\right)=r_{1}\right)\;ds=\sum_{\tilde{m}=m+1}^{\infty }\sum_{r_{2}\in \nabla_{\tilde{m}}}p\left(R_{x_{0}}\left(t\right)\right)=r_{2}).$$

**Proof.**  Fix m∈N. Let ${\left(R_{x_{0},\nabla_{m}}\left(t\right)\right)}_{t\geq 0}$ be the same process as ${\left(R_{x_{0}}\left(t\right)\right)}_{t\geq 0},$ but with the restriction that all propensities of states $r\in \cup_{\tilde{m}=m+1}^{\infty }\nabla_{\hat{m}}$ are zero. Since in the reaction counts case, states only transition forward, we have that,

(15) $$p\left(R_{x_{0}}\left(t\right)=r\right)=p\left(R_{x_{0},\nabla_{m}}\left(t\right)=r\right),\;\mathrm{for}\;\mathrm{all}\;r\in \cup_{\tilde{m}=0}^{m}\nabla_{\tilde{m}}.$$

That is, the probability of both processes are the same for states leading up to the states in $\nabla_{m}.$ However, since the process $R_{x_{0},\nabla_{m}}\left(t\right)$ does not evolve past $\nabla_{m}$, by the conservation of probability we have that,

(16) $$\begin{array}{cc} \sum_{\tilde{m}=0}^{m}\sum_{r\in \nabla_{\tilde{m}}}p\left(R_{x_{0},\nabla_{m}}\left(t\right)=r\right)+\sum_{r_{1}\in \nabla_{m}}\sum_{n=1}^{N_{r}}\alpha_{n}\left(x_{0},r_{1}\right)\;\int_{0}^{t}p\left(R_{x_{0},\nabla_{m}}\left(s\right)=r_{1}\right)\;ds & =1. \end{array}$$

Substituting in Equation (15) for states which appear before $\nabla_{m+1}$ reduces the above expression to,

(17) $$\begin{array}{cc} \sum_{\tilde{m}=0}^{m}\sum_{r\in \nabla_{\tilde{m}}}p\left(R_{x_{0}}\left(t\right)=r\right)+\sum_{r_{1}\in \nabla_{m}}\sum_{n=1}^{N_{r}}\alpha_{n}\left(x_{0},r_{1}\right)\;\int_{0}^{t}p\left(R_{x_{0}}\left(s\right)=r_{1}\right)\;ds & =1. \end{array}$$

By the conservation of probability,

$$\sum_{\tilde{m}=0}^{\infty }\sum_{r_{2}\in \nabla_{\tilde{m}}}p\left(R_{x_{0}}\left(t\right)=r_{2}\right)=1,$$

hence, substituting this into the right-hand side of Equation (17) and subtracting the like terms gives,

$$\sum_{r_{1}\in \nabla_{m}}\sum_{n=1}^{N_{r}}\alpha_{n}\left(x_{0},r_{1}\right)\;\int_{0}^{t}p\left(R_{x_{0}}\left(s\right)=r_{1}\right)\;ds=\sum_{\tilde{m}=m+1}^{\infty }\sum_{r_{2}\in \nabla_{\tilde{m}}}p\left(R_{x_{0}}\left(t\right)=r_{2}\right).$$

 ☐

Lemma 1 is an alternative formulation of the principle behind the Finite State Projection method. We extend on this result and can show that for a desired error ε∈(0,1), there exists a subset of the state space Λ which will produce an approximation with the desired error.

**Theorem** **2.** *For $x_{0}\in \Omega ,$ let ${\left(R_{x_{0}}\left(t\right)\right)}_{t\geq 0}$ be the reaction counts Kurtz process *(5)*. For ε>0 there exists m∈N such that,*

$$\sum_{\tilde{m}=m+1}^{\infty }\sum_{r\in \nabla_{\tilde{m}}}p\left(R_{x_{0}}\left(t\right)=r\right)<\varepsilon .$$

**Proof.**  The proof is an extension of Lemma 1. We define,

$$\phi_{m}:=\sum_{r_{1}\in \nabla_{m}}\sum_{n=1}^{N_{r}}\alpha_{n}\left(x_{0},r_{1}\right)\;\int_{0}^{t}p\left(R_{x_{0}}\left(s\right)=r_{1}\right)\;ds,$$

and,

$$\psi_{m+1}:=\sum_{\tilde{m}=m+1}^{\infty }\sum_{r_{2}\in \nabla_{\tilde{m}}}p\left(R_{x_{0}}\left(t\right)=r_{2}\right),$$

the left- and right-hand terms of Equation (14). Then for m∈N, Lemma 1 can be reformulated to state,

$$\begin{array}{cc} \phi_{m} & =\psi_{m+1}; \end{array}$$

separating the $\nabla_{m+1}$ states from $\psi_{m+1}$ gives us,

$$\begin{array}{c} =\sum_{r\in \nabla_{m+1}}p\left(R_{x_{0}}\left(t\right)=r\right)+\underset{:=\psi_{m+2}}{\underset{︸}{\sum_{\tilde{m}=m+2}^{\infty }\sum_{r_{2}\in \nabla_{\tilde{m}}}p\left(R_{x_{0}}\left(t\right)=r_{2}\right)}},\\ =\sum_{r\in \nabla_{m+1}}p\left(R_{x_{0}}\left(t\right)=r\right)+\psi_{m+2}. \end{array}$$

Combining the last and first term in the equalities gives us,

$$\begin{array}{cc} \psi_{m+1} & =\sum_{r\in \nabla_{m+1}}p\left(R_{x_{0}}\left(t\right)=r\right)+\psi_{m+2}. \end{array}$$

Rearranging the equality reduces to, $\psi_{m+2}-\psi_{m+1}<0.$ We have shown that $\psi_{•}$ is monotonically decreasing. By definition, $\psi_{•}$ has the properties that $\psi_{1}=1.0-p\left(R_{x_{0}}\left(t\right)=0\right)$ and $\lim_{m\to \infty }\psi_{m}=0.$ Hence, there exists an *m* such that $\psi_{m}<\varepsilon .$ ☐

Theorem 2 is an alternative proof for the fact that, when studying transient dynamics (finite time) of the CME for an arbitrary precision, one can always find a finite state space to project the CME onto. The key structure that we used to prove this is that the state space of reaction counts has a natural partitioning and that the probability only flows forward. This gave us the monotonicity needed to prove that state space truncation of the CME is well-posed. In the next section, we further decompose each state in the reaction counts state space into paths over the state space.

## 4. Paths

**Definition** **2.** *For m∈{2,3,…}, a vector $g=\left(g_{1},\ldots ,g_{m}\right)\in \Lambda^{m}$ is said to be an admissible path if for every index i∈{2,…,m} there exists an $n\in \{1,\ldots ,N_{r}\}$ such that $g_{i}=g_{i-1}+1_{n},$ where $1_{n}$ is the identity vector with one in the nth position.*

**Definition** **3.** *For m∈{2,3,…}, we denote the set of all admissible paths of length m by,*

$$\gamma_{m}:=\left\{g\in \Lambda^{m}\;:\;g\;\mathrm{is}\;\mathrm{admissible}\;\right\}$$

**Definition** **4.** *For a point r∈Λ, we denote the set of all admissible paths of length ${∥r∥}_{1}+1$ which start at the origin 0 and end at the state r by,*

$$G\left(r\right):=\left\{g\in \gamma_{{∥r∥}_{1}+1}\;:\;g_{1}=0\;\mathrm{and}\;g_{{∥r∥}_{1}+1}=r\right\}.$$

### 4.1. Path Chains

**Figure 2:**
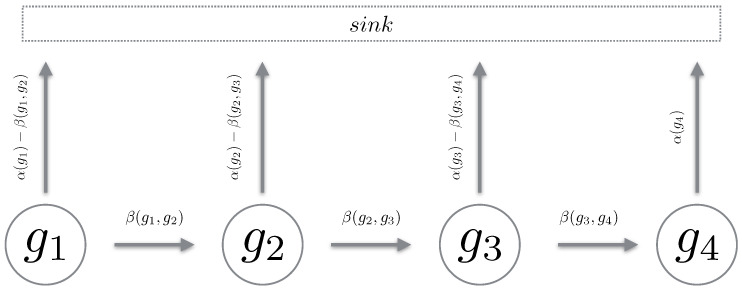
The cartoon above depicts the generator of a path chain. With α(·) being the total outward propensity and β(·,·) the propensity to transition to the next state in the chain. All reactions leading away from the chain are directed into the sink state.

We define a Markov chain over a path in the reaction counts state space. The chain must be such that it mimics a “realisation” over the reaction state space. Using Proposition 1, we define a path chain over an admissible path in the reaction state space. It is important to note that a path chain is a continuous-time Markov chain (CTMC) [19], where all reactions which transition the state off the path of the chain are accrued into a sink state. For brevity, we simply remove this component and only describe the states of interest (see Figure 2). Also, given all chains are from the same reaction counts setting, we omit explicitly stating $x_{0}$ in the propensity functions. We now define a path chain.

**Definition** **5.** *For m∈N,$g\in \gamma_{m},$ and C∈(0,1], we define a path chain to be the stochastic process ${\left(X_{g,C}\left(t\right)\right)}_{t\geq 0},$ with state space {g∪{sink}} and the probability distribution given by,*

(18) $$p\left(X_{g,C}\left(t\right)=g_{1}\right):=e^{-\alpha (g_{1})\;t}C,$$

(19) $$p\left(X_{g,C}\left(t\right)=g_{k}\right):=\beta \left(g_{k-1},g_{k}\right)\;\int_{0}^{t}e^{-\alpha \left(g_{k}\right)\;\left(t-s\right)}\;p\left(X_{g,C}\left(s\right)=g_{k-1}\right)\;ds,$$

*for k∈{2,…,m}. Then to conserve probability,*

$$p\left(X_{g,C}\left(t\right)=g_{k}\right)=\mathrm{sink}):=1.0-\sum_{g_{k}\in g}p\left(X_{g,C}\left(t\right)=g_{k}\right).$$

*The propensity function α is inherited from Equation *(9)* and $\beta \left(g_{k},g_{k+1}\right):=\alpha_{n}\left(g_{k}\right),$ where n is the reaction index which transitions the state $g_{i}$ to $g_{i+1}.$*

Before stating the new proposition, let us recall the decompositions performed so far. Firstly, we showed that every state in the species state space can be decomposed into multiple reaction states in the reaction count setting. That is, a population state can be decomposed into all the different ways in which it can be visited. We then ventured further and showed that a path on the species counts state space corresponds to a path with only forward stepping transitions in the reaction counts state space. Now, we extend this further by showing that any state in the reaction counts state space can be decomposed into the sum of all independent path chains which start at the origin and end in that state.

**Proposition** **2.** *For $x_{0}\in \Omega ,$ let ${\left(R_{x_{0}}\left(t\right)\right)}_{t\geq 0}$ be the Reaction counts Kurtz process *(5)*.*

*For r∈Λ\0, let*

$$G\left(r\right):=\left\{g\in \gamma_{{∥r∥}_{1}+1}\;:\;g_{1}=0\;and\;g_{{∥r∥}_{1}+1}=r\right\},$$

*be the set of all admissible paths of length ${∥r∥}_{1}+1$ which start at 0 and ending at r (Definition 4).*

*Then,*

(20) $$p\left(R_{x_{0}}\left(t\right)=r\right)=\sum_{g\in G(r)}p\left(X_{g,1}\left(t\right)=r\right).$$

**Proof.**  The Proposition will be proved using mathematical induction. Firstly, we show the base case; that every state which is reachable by one step from the origin satisfies Equation (20). Let $r=I_{n},$ with $n\in \{1,\ldots ,N_{r}\}.$ Applying Proposition 1 we know that,

(21) $$p\left(R_{x_{0}}\left(t\right)=I_{n}\right)=\alpha_{n}\left(0\right)\int_{0}^{t}e^{-a\left(I_{n}\right)\left(t-s\right)}\;p\left(R_{x_{0}}\left(s\right)=0\right)\;ds.$$

Now we consider the path chains leading to $I_{n}.$ The state can only be reached in one way, hence, we have that $G\left(I_{n}\right)=\left\{g=\left(0,I_{n}\right)\right\}.$ Now calculating the probability of the evolution of a path chain (Definition 5) over $g\in G(I_{n})$ gives us,

(22) $$p\left(X_{g,1.0}\left(t\right)=I_{n}\right):=\beta \left(0,I_{n}\right)\int_{0}^{t}e^{-\alpha \left(I_{n}\right)\;\left(t-s\right)}\;p\left(X_{g,1}\left(s\right)=0\right)\;ds.$$

Given the two equations above match, we can conclude that for $r\in \nabla_{1},$

$$p\left(R\left(t\right)=r\right)=\sum_{g\in G(r)}p\left(X_{g,1}\left(t\right)=r\right).$$

Now we perform the inductive step. Fix m>1. Assume that for $\overset{ˇ}{r}\in \nabla_{m},$

(23) $$p\left(R_{x_{0}}\left(t\right)=\overset{ˇ}{r}\right)=\sum_{g\in G(\overset{ˇ}{r})}p\left(X_{g,1}\left(t\right)=\overset{ˇ}{r}\right).$$

Then for $r\in \nabla_{m+1},$ Proposition 1 states that,

$$\begin{array}{cc} p(R(t)=r)= & \sum_{n=1}^{N_{r}}\alpha_{n}\left(r-I_{n}\right)\;\int_{0}^{t}e^{-\alpha (r)\;(t-s)}\;p\left(R_{x_{0}}\left(s\right)=r-I_{n}\right)\;ds, \end{array}$$

using the inductive assumption Equation (23) on the right-hand side inside the integral,

$$\begin{array}{cc} = & \sum_{n=1}^{N_{r}}\alpha_{n}\left(r-I_{n}\right)\;\int_{0}^{t}e^{-\alpha (r)\;(t-s)}\sum_{g\in G(r-I_{n})}p\left(X_{g,1}\left(s\right)=r-I_{n}\right)\;ds. \end{array}$$

Rewriting the propensities in the path propensity notation gives,

$$\begin{array}{cc} = & \sum_{n=1}^{N_{r}}\beta \left(r-I_{n},r\right)\;\int_{0}^{t}e^{-\alpha (r)\;(t-s)}\sum_{g\in G(r-I_{n})}p\left(X_{g,1}\left(s\right)=r-I_{n}\right)ds,\\ = & \sum_{n=1}^{N_{r}}\;\sum_{g\in G(r-I_{n})}\beta \left(r-I_{n},r\right)\int_{0}^{t}e^{-\alpha (r)\;(t-s)}\;p\left(X_{g,1}\left(s\right)=r-I_{n}\right)\;ds. \end{array}$$

Since all paths in G(r) are paths from the set $\cup_{n=1}^{N_{r}}G\left(r-I_{n}\right),$ with *r* as the last state, the two summations reduce to give,

$$\begin{array}{cc} = & \sum_{\hat{g}\in G\left(r\right)}\beta \left({\hat{g}}_{m},r\right)\int_{0}^{t}e^{-\alpha (r)\;(t-s)}\;p\left(X_{\hat{g},1}\left(s\right)={\hat{g}}_{m}\right)\;ds. \end{array}$$

By Definition 5, the term above captures all the paths in G(r), therefore,

$$\begin{array}{cc} = & \sum_{\hat{g}\in G\left(r\right)}p\left(X_{\hat{g},1}\left(s\right)=r\right)\;ds. \end{array}$$

We have shown that given Equation (23) holds on $\nabla_{m},$ then Equation (23) also holds for $\nabla_{m+1}.$ Hence, by mathematical induction, for any r∈Λ,

$$p\left(R_{x_{0}}\left(t\right)=r\right)=\sum_{g\in G(r)}p\left(X_{g,1}\left(t\right)=r\right)\;ds.$$

 ☐

We have shown that each state in the reaction counts CME can be decomposed into the framework of “path chains” that we have defined. In essence, we have shown that the path chains are trajectories of the reaction counts Kurtz process. Even though at first glance the result is not a startling revelation, the real novelty lies in how we derived the probability distributions for these trajectories. By carefully decomposing the state space and concurrently deriving the corresponding probability distributions, we were able to give an analytical form for the probability of individual trajectories of the Kurtz process. Using the probability distribution given over the path chains in Definition 5, we can derive the classical stochastic simulation algorithm [20]. Using the path chains framework we have introduced so far, we can build realisations for applications such as: Hitting time problems, rare event problems, observable problems, etc. In the following section, we further build on the properties of path chains by proposing a method for estimating the path probabilities of trajectories.

### 4.2. Gated- and Un-Gated Path Chains

Given a path or trajectory, we define the path probability to be the probability distribution of the last state in the chain. We can use Definition 5 to analytically compute the probability of any path. However, we can exploit the properties of the path chains further and derive some simple estimates for the path probability. We borrow the notion of gating and un-gating introduced by Sunkara (2013) [21], and show how these can be used to calculate estimates for the path probabilities.

The concept of gating is mathematically tedious to prove, however, the concept is fairly simple. Given a chain *g* of length m, gating this chain at position j<m means that we set the propensity of leaving state $g_{j}$ to zero. Hence, the probability will simply flow into state $g_{j}$ and remain there (hence, the term “gating”). Then we can “un-gate” the state $g_{j},$ which means that we reset the time to zero, set the initial probability at state $g_{j}$ to the accumulated probability from the gating, and then allow the process to continue. The reason we are interested in this notion of gating and un-gating is that for path chains, it happens so that gating and un-gating the chains gives an upper bound for the original path probability. We will now prove that this is indeed the case.

**Definition** **6.** *For an admissible path $g\in \gamma_{m},$ we define a truncated path $g^{j}:=\left(g_{j},g_{j+1},\ldots ,g_{m}\right),$ where the first j−1 terms of the path are ignored.*

**Definition** **7.** *Fix Δt<∞ and $x_{0}\in \Omega .$ Let $g\in \gamma_{m}$ be a path of length m+1. We define ${\left(X_{g,C}\left(t\right)\right)}_{t\in [0,\Delta t]}$ to be a path chain over g in the time interval [0,Δt]. Then for j∈{2,…,m} we define an un-gated path chain over $g^{j}$ to be given by:*

(24) $$p\left(X_{g^{j},C}\left(t\right)=g_{k}\right):=\beta \left(g_{k-1},g_{k}\right)\;\int_{0}^{t}e^{-\alpha \left(g_{k}\right)\;\left(t-s\right)}\;p\left(X_{g,C_{j}}\left(s\right)=g_{k-1}\right)\;ds,$$

*for k∈{j+1,…,m} and,*

$$p\left(X_{g^{j},C}\left(t\right)=g_{j}\right):=e^{-\alpha (g_{j})\;t}\;C_{j}$$

*where,*

$$C_{j}:=\beta \left(g_{j-1},g_{j}\right)\int_{0}^{\Delta t}p\left(X_{g,C}\left(s\right)=g_{j-1}\right).$$

*To conserve probability,*

$$p\left(X_{g^{j},C}\left(t\right)=g_{k}\right)=\mathrm{sink}):=1.0-\sum_{g_{k}\in g^{j}}p\left(X_{g,C}\left(t\right)=g_{k}\right).$$

**Figure 3:**
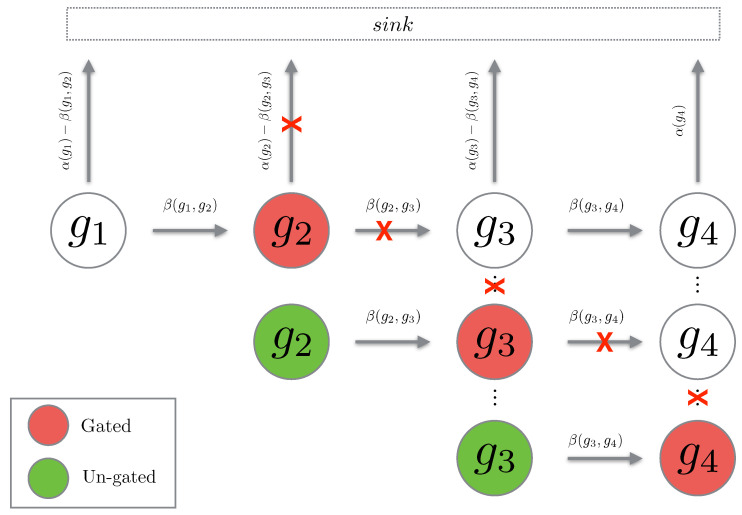
The cartoon above depicts the cascade of gating being performed on a path chain. When a state is gated, the propensities leaving the state are set to zero (depicted with a red cross). When the state is un-gated, the propensities are reintroduced.

We see that the un-gated path chain is a normal path chain with the initial probability reset to the total probability which has flown into the gated state (see Figure 3). We now show that gating and un-gating over a path gives an upper bound for the path probability. First we consider the case of a single gating and un-gating at the position $g_{2}.$

**Lemma** **2.** *Fix $g\in \gamma_{m}$ and Δt>0. Given two path chains ${\left(X_{g,C}\left(t\right)\right)}_{t\in [0,\Delta t]}$ and ${\left(X_{g^{2},C_{2}}\left(t\right)\right)}_{t\in [0,\Delta t]},$ it follows that for all t∈[0,Δt],*

$$\int_{0}^{t}p\left(X_{g,C}\left(s\right)=2\right)\;dt\leq \int_{0}^{t}p\left(X_{g^{2},C_{2}}\left(s\right)=2\right)\;dt,$$

*and for j∈{3,…,m}*

$$p\left(X_{g,C}\left(t\right)=g_{j}\right)\leq p\left(X_{g^{2},C_{2}}\left(t\right)=g_{j}\right).$$

**Proof.**  We break down the proof into two steps. First we investigate the bounds formed by gating at the second step, and then we prove the bound over the remaining states in the chain. We begin by showing that for t∈[0,Δt],

(25) $$\int_{0}^{t}p\left(X_{g,C}\left(s\right)=2\right)\;dt\leq \int_{0}^{t}p\left(X_{g^{2},C_{2}}\left(s\right)=2\right)\;dt.$$

From the definition of the path chain (Definition 5), the solution for the probability of being in state $g_{2}$ at time *t* is given by,

(26) $$p\left(X_{g,C}\left(t\right)=g_{2}\right)=\frac{C\;\beta (g_{1},g_{2})}{\alpha \left(g_{1}\right)-\alpha \left(g_{2}\right)}\;\left(e^{-\alpha (g_{2})t}-e^{-\alpha (g_{1})t}\right).$$

Using the solution above, we can calculate the probability which would accumulate in $g_{2}$ if the chain was gated at $g_{2},$

(27) $$C_{2}:=\frac{C\;\beta (g_{1},g_{2})}{\alpha (g_{1})}\;\left(1-e^{-\alpha (g_{1})\Delta t}\right).$$

Substituting the above terms into the definition of the probability of being is state $g_{2}$ at time *t* in the un-gated path chain ${\left(X_{g^{2},C_{2}}\left(t\right)\right)}_{t\in [0,\Delta t]}$ gives us,

(28) $$p\left(X_{g^{2},C_{2}}\left(t\right)=g_{2}\right)=\frac{C\;\beta (g_{1},g_{2})}{\alpha (g_{1})}\;\left(1-e^{-\alpha (g_{1})\Delta t}\right)\;e^{-\alpha (g_{2})t}.$$

Taking the integral of Equations (26) and (28) over the interval [0,t]⊆[0,Δt] gives us,

(29) $$\begin{array}{cc} \int_{0}^{t}p\left(X_{g,C}\left(s\right)=g_{2}\right)\;ds & =\frac{C\;\beta (g_{1},g_{2})}{\left(\alpha \left(g_{1}\right)-\alpha \left(g_{2}\right)\right)\;\alpha \left(g_{1}\right)\;\alpha \left(g_{1}\right)}\left(\alpha \left(g_{2}\right)\left(e^{-\alpha (g_{1})\;t}-1\right)-\alpha \left(g_{1}\right)\left(e^{-\alpha (g_{2})\;t}-1\right)\right), \end{array}$$

(30) $$\begin{array}{cc} \int_{0}^{t}p\left(X_{g^{2},C_{2}}\left(s\right)=g_{2}\right)\;ds & =\frac{C\;\beta (g_{1},g_{2})}{\alpha \left(g_{1}\right)\;\alpha \left(g_{2}\right)}\;\left(1-e^{-\alpha (g_{1})\Delta t}\right)\;\left(1-e^{-\alpha (g_{2})t}\right). \end{array}$$

To simplify notation, let us define,

$$\hat{\phi }\left(t\right):=\int_{0}^{t}p\left(X_{g^{2},C_{2}}\left(s\right)=g_{2}\right)\;ds\;\mathrm{and}\;\phi \left(t\right):=\int_{0}^{t}p\left(X_{g,C}\left(s\right)=g_{2}\right)\;ds.$$

Then the difference between $\hat{\phi }\left(t\right)$ and ϕ(t) reduces to,

(31) $$\begin{array}{cc} \hat{\phi }\left(t\right)-\phi \left(t\right) & =\frac{C\;\beta (g_{1},g_{2})}{\alpha \left(g_{1}\right)\;\alpha \left(g_{2}\right)}\;\left(1-e^{-\alpha (g_{1})\Delta t}\right)\;\left(1-e^{-\alpha (g_{2})t}\right)\\ & \;-\frac{C\;\beta (g_{1},g_{2})}{\left(\alpha \left(g_{1}\right)-\alpha \left(g_{2}\right)\right)\;\alpha \left(g_{1}\right)\;\alpha \left(g_{1}\right)}\left(\alpha \left(g_{2}\right)\left(e^{-\alpha (g_{1})\;t}-1\right)-\alpha \left(g_{1}\right)\left(e^{-\alpha (g_{2})\;t}-1\right)\right). \end{array}$$

Since $e^{-\alpha (g_{1})\Delta t}\leq e^{-\alpha (g_{1})t}$ for all t∈[0,Δt], we attain the lower bound,

(32) $$\begin{array}{c} \geq \frac{C\;\beta (g_{1},g_{2})}{\alpha \left(g_{1}\right)\;\alpha \left(g_{2}\right)}\;\left(1-e^{-\alpha (g_{1})t}\right)\;\left(1-e^{-\alpha (g_{2})t}\right)\\ \;-\frac{C\;\beta (g_{1},g_{2})}{\left(\alpha \left(g_{1}\right)-\alpha \left(g_{2}\right)\right)\;\alpha \left(g_{1}\right)\;\alpha \left(g_{1}\right)}\left(\alpha \left(g_{2}\right)\left(e^{-\alpha (g_{1})\;t}-1\right)-\alpha \left(g_{1}\right)\left(e^{-\alpha (g_{2})\;t}-1\right)\right). \end{array}$$

We show that the right-hand side of the equation above is always positive. We define the right hand-side of Equation (32) as a function,

(33) $$\begin{array}{c} \Psi \left(g_{1},g_{2},t\right):=\frac{C\;\beta \left(g_{1},g_{2}\right)\;\left(e^{-\alpha (g_{2})t}\alpha \left(g_{2}\right)-e^{-\alpha (g_{1})t}\alpha \left(g_{1}\right)+e^{-(\alpha \left(g_{1}\right)+\alpha \left(g_{2}\right))t}\left(\alpha \left(g_{1}\right)-\alpha \left(g_{2}\right)\right)\right)}{\left(\alpha \left(g_{1}\right)-\alpha \left(g_{2}\right)\right)\;\alpha \left(g_{1}\right)\;\alpha \left(g_{2}\right)}. \end{array}$$

We notice that the function Ψ has the property,

$$\Psi \left(g_{1},g_{2},t\right)=\Psi \left(g_{2},g_{1},t\right).$$

Firstly, if we consider the case $\alpha \left(g_{1}\right)=\alpha \left(g_{2}\right),$ we can see that $\psi (g_{1},g_{2},t)=0.$ Using the symmetry of Ψ, we only need to consider the case $\alpha \left(g_{1}\right)<\alpha \left(g_{2}\right).$ Then it follows that,

$$\begin{array}{cc} \Psi (g_{1},g_{2},t) & =\frac{C\;\beta \left(g_{1},g_{2}\right)\;\left(e^{-\alpha (g_{2})\Delta t}\alpha \left(g_{2}\right)-e^{-\alpha (g_{1})t}\alpha \left(g_{1}\right)+e^{-(\alpha \left(g_{1}\right)+\alpha \left(g_{2}\right))t}\left(\alpha \left(g_{1}\right)-\alpha \left(g_{2}\right)\right)\right)}{\left(\alpha \left(g_{1}\right)-\alpha \left(g_{2}\right)\right)\;\alpha \left(g_{1}\right)\;\alpha \left(g_{2}\right)}, \end{array}$$

replacing $e^{-\alpha (g_{1})t}\alpha \left(g_{1}\right)$ with $e^{-\alpha (g_{1})t}\alpha \left(g_{2}\right)$ gives us,

$$\begin{array}{c} \geq \frac{C\;\beta \left(g_{1},g_{2}\right)\;\left(\left(e^{-\alpha (g_{2})t}-e^{-\alpha (g_{1})t}\right)\alpha \left(g_{2}\right)+e^{-(\alpha \left(g_{1}\right)+\alpha \left(g_{2}\right))t}\left(\alpha \left(g_{1}\right)-\alpha \left(g_{2}\right)\right)\right)}{\left(\alpha \left(g_{1}\right)-\alpha \left(g_{2}\right)\right)\;\alpha \left(g_{1}\right)\;\alpha \left(g_{2}\right)},\\ =C\;\beta \left(g_{1},g_{2}\right)\;\left(\underset{>0}{\underset{︸}{\frac{\left(e^{-\alpha (g_{2})t}-e^{-\alpha (g_{1})t}\right)}{\left(\alpha \left(g_{1}\right)-\alpha \left(g_{2}\right)\right)\;\alpha \left(g_{1}\right)}}}+\underset{>0}{\underset{︸}{\frac{e^{-(\alpha \left(g_{1}\right)+\alpha \left(g_{2}\right))\Delta t})}{\alpha \left(g_{1}\right)\;\alpha \left(g_{2}\right)}}}\right),\\ \geq 0. \end{array}$$

Hence, the right-hand side of Equation (33) is positive. So it follows that for t∈[0,Δt],

$$\int_{0}^{t}p\left(X_{g,C}\left(s\right)=2\right)\;ds\leq \int_{0}^{t}p\left(X_{g^{2},C_{2}}\left(s\right)=2\right)\;ds.$$

We have shown that the outflow of probability in the un-gated chain is more than in the original path chain. We now show that this phenomenon continues all the way down the chain. We prove this using mathematical induction. First, let us consider the base case j=3. By the definition of path chains,

$$\begin{array}{cc} p\left(X_{g,C}\left(t\right)=g_{3}\right)-p\left(X_{g^{2},C_{2}}\left(t\right)=g_{3}\right) & =\beta \left(g_{2},g_{3}\right)\int_{0}^{t}e^{-\alpha \left(g_{3}\right)\left(t-s\right)}\left(p\left(X_{g,C}\left(s\right)=g_{2}\right)-p\left(X_{g^{2},C_{2}}\left(s\right)=g_{2}\right)\right)\;ds,\\ & \leq \beta \left(g_{2},g_{3}\right)\;\int_{0}^{t}\left(p\left(X_{g,C}\left(s\right)=g_{2}\right)-p\left(X_{g^{2},C_{2}}\left(s\right)=g_{2}\right)\right)\;ds, \end{array}$$

applying condition (25) gives us that,

$$\begin{array}{c} \leq 0. \end{array}$$

Hence, for t∈[0,Δ],

$$p\left(X_{g,C}\left(t\right)=g_{3}\right)\leq p\left(X_{g^{2},C_{2}}\left(t\right)=g_{3}\right).$$

We now build the inductive step. Fix k∈{4,…,m}, assume that, for all t∈[0,Δt],

(34) $$p\left(X_{g,C}\left(t\right)=g_{k}\right)\leq p\left(X_{g^{2},C_{2}}\left(t\right)=g_{k}\right).$$

Then by the definition of path chains,

$$\begin{array}{cc} p\left(X_{g,C}\left(t\right)=g_{k+1}\right)-p\left(X_{g^{2},C_{2}}\left(t\right)=g_{k+1}\right) & =\beta \left(g_{k},g_{k+1}\right)\int_{0}^{t}e^{-\alpha \left(g_{k+1}\right)\left(t-s\right)}\left(p\left(X_{g,C}\left(s\right)=g_{k}\right)-p\left(X_{g^{2},C_{2}}\left(s\right)=g_{k}\right)\right)\;ds,\\ & \leq \beta \left(g_{k},g_{k+1}\right)\;\int_{0}^{t}\left(p\left(X_{g,C}\left(s\right)=g_{k}\right)-p\left(X_{g^{2},C_{2}}\left(s\right)=g_{k}\right)\right)\;ds, \end{array}$$

applying the inductive assumption Equation (34) gives us that,

$$\begin{array}{c} \leq 0. \end{array}$$

Hence, by mathematical induction, for j∈{3,…,m} and t∈[0,Δt], the probabilities of the un-gated chain bound the probability on the original path chain, that is,

$$p\left(X_{g,C}\left(t\right)=g_{j}\right)\leq p\left(X_{g^{2},C_{2}}\left(t\right)=g_{j}\right).$$

 ☐

The intuition behind Lemma 2 is that once we gate and un-gate a state, the un-gated chain has more probability in it than the regular path chain beyond the gated state. Since the dynamics of the path chain beyond the gated state are not changed, we would expect that the un-gated chain (having more probability) would be an upper bound for the original path chain. The proof, as seen above, is sadly tedious. We now consider a cascade of gating and un-gating, that is, we gate and un-gate as we transition through the path chain. We prove that this gives an upper bound for the path probabilities.

### 4.3. Cascade of Gating and Un-Gating

We now set up a cascade of gating and un-gating at ever state of the path chain. We achieve this by defining the starting probability of the un-gating. Previously, an un-gated path chain at position *j* had a initial probability of,

$$C_{j}:=\beta \left(g_{j-1},g_{j}\right)\int_{0}^{\Delta t}p\left(X_{g,C}\left(s\right)=g_{j-1}\right)\;ds.$$

We now introduce a recurrent initial probability,

(35) $${\tilde{C}}_{j}:=\beta \left(g_{j-1},g_{j}\right)\int_{0}^{\Delta t}p\left(X_{g^{j-1},{\tilde{C}}_{j-1}}\left(s\right)=g_{j-1}\right)\;ds,$$

for j∈{2,…,m−1}. Using this new recursive initial probability, we will build a sequence of path chains and using the idea of gating and un-gating at each transition of the path chain, we can construct an upper bound for the path probability.

**Theorem** **3.** *Fix $g\in \gamma_{m}$ and Δt>0. Let ${\left(X_{g,C}\left(t\right)\right)}_{t\in [0,\Delta t]}$ be a path chain over g. We recursively define a set of un-gated chains, $\{{\left(X_{g^{j},{\tilde{C}}_{j}}\left(t\right)\right)}_{t\in [0,\Delta t]}:j\in \left\{2,\ldots ,m-2\right\}\}.$ Then for k∈{3,…,m−1},*

$$\int_{0}^{\Delta t}p\left(X_{g,C}\left(s\right)=g_{k}\right)\;ds\leq \int_{0}^{\Delta t}p\left(X_{g^{k-1},{\tilde{C}}_{k-1}}\left(s\right)=g_{k}\right)\;ds.$$

**Proof.**  We prove this result by repeatedly applying Lemma 2. Fix k∈{3,…,m−1}, then by the first clause of Lemma 2 we have that,

$$\begin{array}{cc} \int_{0}^{\Delta t}p\left(X_{g^{k-1},{\tilde{C}}_{k-1}}\left(s\right)=g_{k}\right)\;ds & \geq \int_{0}^{\Delta t}p\left(X_{g^{k-2},{\tilde{C}}_{k-2}}\left(s\right)=g_{k}\right)\;ds, \end{array}$$

since $g_{k}$ is the third state of the chain $g^{k-2},$ we can apply the second clause of Lemma 2 to get,

$$\begin{array}{c} \geq \int_{0}^{\Delta t}p\left(X_{g^{k-3},{\tilde{C}}_{k-3}}\left(s\right)=g_{k}\right)\;ds, \end{array}$$

stepping backward in the chain using Lemma 2, we reduce down to,

$$\begin{array}{c} \vdots \\ \geq \int_{0}^{\Delta t}p\left(X_{g,C}\left(s\right)=g_{k}\right)\;ds. \end{array}$$

 ☐

In summary, by gating and un-gating along a path chain we can easily construct estimates for the probability of the trajectories of interest. We now consider an example where we build a path chain of the birth-death process introduced in Example 1, to show how to compute the gating and un-gating steps.

**Example** **2.** *Let us consider the birth-death process introduced in Example 1, with the parameters $x_{0}=2,c_{b}=1.0,$ and $c_{d}=0.1.$ We will now construct the path probability of the chain:*

$$g=\left((0,0),(1,0),(1,1),(1,2),(1,3)\right).$$

*The generator for this path chain is given by,*

$$A^{*}=[\begin{array}{cccccc} \frac{\left(0,0\right)}{-c_{b}-2\;c_{d}} & \frac{\left(1,0\right)}{0} & \frac{\left(1,1\right)}{0} & \frac{\left(1,2\right)}{0} & \frac{\left(1,3\right)}{0} & \frac{sink}{0}\\ c_{b} & -c_{b}-3\;c_{d} & 0 & 0 & 0 & 0\\ 0 & -3\;c_{d} & -c_{b}-2\;c_{d} & 0 & 0 & 0\\ 0 & 0 & 2\;c_{d} & -c_{b}-c_{d} & 0 & 0\\ 0 & 0 & 0 & c_{d} & 0 & 0\\ 2\;c_{d} & c_{b} & c_{b} & c_{b} & 0 & 0 \end{array}].$$

*We can solve for $p(X_{g},1\left(t\right)=\left(1,3\right))$ by simply taking the matrix exponential at time t and applying it to the respective initial probability. Now we will construct the gating and un-gating approximation. This approximation is calculated using the following recursive equation: Let m be the number of states in the chain g, then,*

$$u_{n}\left(t\right)=\frac{\beta (g_{n-1},g_{n})}{\alpha (g_{n-1})}\;\left(1.0-e^{-\alpha (g_{n-1})\;t}\right)\;u_{n-1}\left(t\right),$$

**Figure 4:**
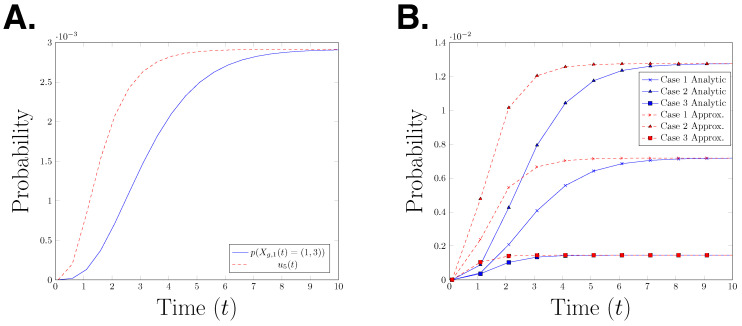
(**A**) Graph of the path probability $p(X_{g,1}\left(t\right)=\left(1,3\right))$ and the upper bound of the path probability $u_{5}\left(t\right)$ for the time interval t∈[0,10]. (**B**) Case 1: $(c_{b}=1.0,c_{d}=0.15),$ Case 2: $(c_{b}=1.0,c_{d}=0.2),$ Case 3: $(c_{b}=2.0,c_{d}=0.15).$ “analytical” refers to the path probability and “approximation” refers to the upper bound of the path probability.

*for n=2,…,m with $u_{1}=1.0.$ The functions α and β are as given in Definition 5. Hence, $u_{5}\left(t\right)$ is the gated and un-gated estimate for the probability $p(X_{g,1}\left(t\right)=\left(1,3\right)).$ In Figure 4A, we plot the true path probability, $p(X_{g,1}\left(t\right)=\left(1,3\right)),$ and the upper bound for the path probability, $u_{5}\left(t\right),$ for time interval t∈[0,10] seconds. The graph shows that $u_{5}\left(t\right)$ is an upper bound for $p(X_{g},1\left(t\right)=\left(1,3\right))$ at any time point. It is interesting to observe that the error between the two functions decreases over time, however it is not clear whether the difference will converge to zero as time goes to infinity. To test whether this behaviour can be observed in other parameter settings, we plot the analytical path probability and its upper bound for three different parameter settings in Figure 4B. We observe the same effect in all three presented cases. This raises the question whether this observation is a structural property or only an artefact of the specific model which is considered here.*

## 5. Conclusions

We began by formulating the reaction counts CME and showing that its forward stepping characteristic yields analytical solutions. Then, with a simple application of the push forward measure, we could prove the existence of solutions of the CME for finite time. We further decomposed the reaction counts state space into independent CTMCs and gave analytical forms and estimates for their path probabilities. Hence, we have derived analytical theory for trajectories which arise from the solutions of the CME. This theory can be used for computing observable estimates of the underlying CTMCs. We can also use the paths to estimate the hitting times and rare event probabilities. A natural future direction would be to investigate if the current framework of the time independent propensities can be translated to the time dependent propensities. An even more challenging task would be, in the context of parameter inference, to reverse time on the trajectories and to cascade probability backwards.
